# Rectus sheath hematoma in an elderly male with anticoagulation therapy—a diagnostic challenge

**DOI:** 10.1093/omcr/omaf213

**Published:** 2025-10-29

**Authors:** Islam Rajab, Ibrahim Sabah, Sakeena Saife, Maram M Abukhalil, Yezin Shamoon, Fayez Shamoon, Henry Alocha

**Affiliations:** St Joseph university medical center, Paterson, NJ, United States; St Joseph university medical center, Paterson, NJ, United States; Department of Medicine, Faculty of Medicine and Health Sciences, An-Najah National University, Nablus, Palestine; Department of Medicine, Faculty of Medicine and Health Sciences, An-Najah National University, Nablus, Palestine; Department of Cardiology, St Joseph's Regional Medical Center, United States; Department of Cardiology, St Joseph's Regional Medical Center, United States; St Joseph university medical center, Paterson, NJ, United States

**Keywords:** rectus sheath hematoma, anticoagulants, hypotension, pelvis, embolization, therapeutic

## Abstract

Rectus sheath hematoma (RSH) is a rare but potentially life-threatening condition, often underdiagnosed due to its non-specific presentation. We report a case of a 71-year-old male admitted for pneumonia, who developed hypotension, urinary retention, and a palpable pelvic mass after receiving low molecular weight heparin (LMWH) for new-onset atrial fibrillation. Ultrasound suggested a pelvic abnormality, and computed tomography angiography (CTA) confirmed a large rectus sheath hematoma with active venous bleeding. The patient underwent successful embolization of the right inferior epigastric artery, resulting in hemodynamic stabilization. This case highlights the importance of maintaining high clinical suspicion for RSH in anticoagulated patients and emphasizes the need for early imaging and multidisciplinary management to prevent complications.

## Introduction

Rectus sheath hematoma (RSH) is an uncommon but clinically significant condition resulting from bleeding into the sheath of the rectus abdominis muscle [1]. The condition typically arises due to vascular rupture of the superior or inferior epigastric arteries, with major risk factors including anticoagulation therapy (present in up to 70% of cases), trauma, vascular fragility, and strenuous physical activity [2]. The incidence of RSH has been increasing in recent years, primarily due to the growing use of anticoagulants, particularly among elderly patients with multiple comorbidities [3]. In severe cases, mortality reaches 4% [1]. RSH may mimic abdominal emergencies, making diagnosis difficult. Rapid diagnosis often relies on bedside ultrasonography, while contrast-enhanced computed tomography (CT) and magnetic resonance imaging (MRI) offer detailed characterization of hematoma size, location, and active bleeding. Several studies report a higher prevalence of RSH in elderly females, particularly those receiving anticoagulation therapy, due to increased abdominal wall laxity and vascular fragility. Here, we present a case of massive RSH causing recurrent hypotension in an anticoagulated elderly male, highlighting the diagnostic and therapeutic challenges of this condition. This case is notable for the massive size of the hematoma, rapid hemodynamic compromise, and its rare complication of bladder outlet obstruction, which together posed a diagnostic and therapeutic challenge.

## Case presentation

A 71-year-old male with a history of benign prostatic hyperplasia (post-TURP 2020), scoliosis, pectus excavatum, peptic ulcer disease, diastolic heart failure (EF 55%), and hypertension presented to the emergency department with a 3–4-day history of shortness of breath, non-productive cough, and fatigue. He was afebrile but hypoxic on arrival and required supplemental oxygen via nasal cannula.

Initial labs revealed leukocytosis and mild hypoxemia. Chest X-ray showed a right lower lobe consolidation, consistent with community-acquired pneumonia. He was admitted and started on antibiotics.

On hospital day three, he developed new-onset atrial fibrillation. Rate control was achieved with intravenous then oral diltiazem. Full-dose low molecular weight heparin (LMWH) was started for stroke prevention.

By day five, the patient developed hypotension (mean arterial pressure [MAP] < 60 mmHg). IV fluids provided transient stabilization. Bladder scan revealed urinary retention, but catheterization attempts failed. Transabdominal ultrasound showed a pelvic mass-like lesion, raising concern for hematoma or abscess.

**Figure 1:**
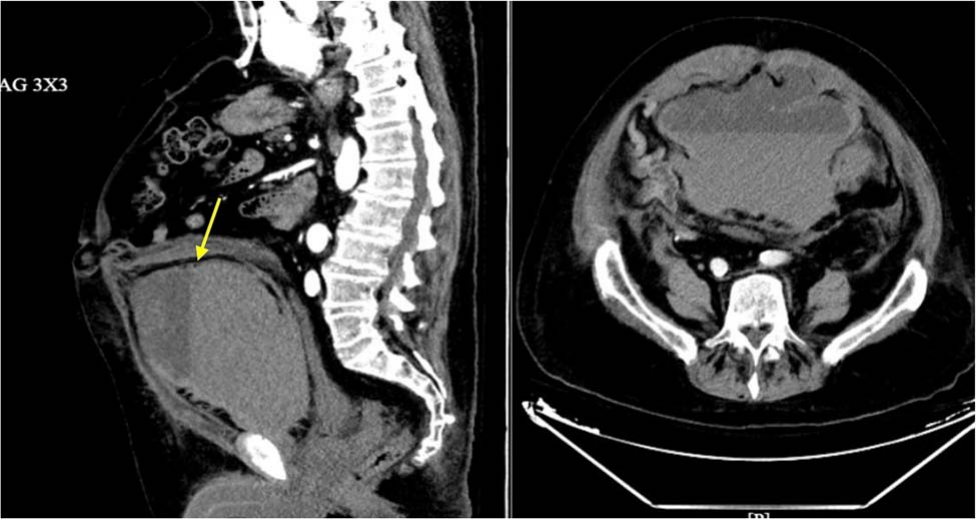
CT angiography of the abdomen and pelvis: Contrast-enhanced CT showing a large, complex hematoma (arrow) arising from the rectus sheath in the anterior pelvis, measuring approximately 14.0 × 19.0 × 15.9 cm. Subtle contrast layering in the delayed venous phase suggests mild active venous bleeding.

A CT angiography of the abdomen and pelvis was performed, revealing a large 14 × 19 × 15.9 cm rectus sheath hematoma with delayed venous contrast pooling (Fig. 1). Interventional radiology was consulted, and the patient underwent urgent embolization of the right inferior epigastric artery. After the procedure, his hemodynamic status stabilized.

## Discussion

This patient’s evolving symptoms delayed diagnosis. Initially admitted with community-acquired pneumonia, he later developed new-onset atrial fibrillation and was started on therapeutic anticoagulation. Within days, he experienced hypotension, urinary retention, and a palpable pelvic mass. These overlapping findings initially led clinicians to suspect sepsis or volume depletion. However, CT angiography revealed a large rectus sheath hematoma (RSH) with active venous bleeding [4].

The hematoma was classified as Grade III—a large, complex, multi-septated collection exceeding 14 cm, causing mass effect and hemorrhage. Grade III hematomas are often linked to hemodynamic instability, intra-abdominal compartment syndrome, and obstructive uropathy, as seen in this patient who developed acute urinary retention due to mass compression.

Risk factors for RSH include advanced age, anticoagulation, female sex, chronic cough, strenuous activity, and prior abdominal surgery. Our patient had several: anticoagulation with low molecular weight heparin, hypovolemia from diuretics, pneumonia-related cough, and structural deformities (pectus excavatum, scoliosis). Diastolic heart failure may have contributed further by increasing capillary fragility.

CT angiography remains the gold standard for diagnosing RSH, offering crucial detail on the size, location, extent of bleeding, and any active extravasation [4]. In this case, multiphasic imaging identified delayed venous bleeding not seen on arterial phase. Embolization of the right inferior epigastric artery led to hemodynamic stabilization.

Literature comparisons highlight both similarities and distinctions. Hatjipetrou et al. described a 75-year-old female with hypotension on warfarin, treated conservatively. Takahashi et al. reported successful embolization in an elderly male on warfarin. Logan’s case involved LMWH and COVID-19. In contrast, our patient had pneumonia, multiple anatomical risk factors, and required urgent intervention. These cases underscore the variability of RSH and the need for tailored management.

Management ranges from conservative (stopping anticoagulation, fluids, monitoring) to surgery or embolization. Embolization is preferred in unstable patients due to its minimally invasive nature and high success rate. Surgery is reserved for embolization failure^10^.

Clinicians should maintain high suspicion for RSH in anticoagulated patients with unexplained hypotension. Prompt imaging and early interventional radiology referral are key. This case highlights the importance of multidisciplinary coordination in achieving positive outcomes. Table 1 summarizes reported RSH cases with their clinical features, risk factors, and outcomes.

**Table 1:** 

Author, Year	Age	Gender	Risk Factor(s)	Presentation	Management	Outcome
Hatjipetro u et al., 2015 [4]	75	Female	Anticoagulation n (warfarin), hypertension	Acute abdominal pain, hypotension	Conservative, fluid resuscitation	Recovered
Cherry & Mueller, 2006 [5]	66	Female	Coughing, anticoagulation	Palpable mass, abdominal pain	Embolization	Recovered
Takahashi et al., 2019 [6]	82	Male	Warfarin use	Hemodynami c instability, anemia	Embolization	Recovered
Logan, 2022 [7]	67	Female	LMWH (therapeutic dose), COVID- positive	Abdominal distension, syncope	Discontinuatio n of LMWH, supportive	Recovered
Almannie & Alkhamis, 2018 [8]	70	Male	Enoxaparin (prophylactic), renal impairment	Hematuria, bladder perforation	Surgical intervention	Recovered

LMWH = Low Molecular Weight Heparin

## Supplementary Material

cover_letter_omaf213
