# Supplementary material for: Rectus sheath hematoma in an elderly male with anticoagulation therapy—a diagnostic challenge
Source: Oxf Med Case Reports. 2025 Oct 29;2025(10):omaf213. doi: 10.1093/omcr/omaf213 (PMC12570027; doi:10.1093/omcr/omaf213)
Supplement: cover_letter_omaf213 [file cover_letter_omaf213.pdf]

Dear Editor,

We are pleased to submit our case report titled “Rectus Sheath Hematoma in an Elderly Male with Anticoagulation Therapy – A Diagnostic Challenge” for consideration in Oxford Medical Case Reports.

This case highlights the importance of early recognition and multidisciplinary management of rectus sheath hematoma in anticoagulated patients.

All authors have approved the manuscript, and it is not under consideration elsewhere.

Sincerely,

Sakeena Saife

On behalf of all co-authors
